# Resolving the Puzzle of *Iris maackii* (Iridaceae): A Morphological Insight into Its Taxonomy

**DOI:** 10.3390/plants12193349

**Published:** 2023-09-22

**Authors:** Eugeny V. Boltenkov

**Affiliations:** Botanical Garden-Institute, Far Eastern Branch, Russian Academy of Sciences, 690024 Vladivostok, Russia; boltenkov@rambler.ru

**Keywords:** China, *Iris laevigata*, *Iris pseudacorus*, morphological characters, morphometry, multivariate analysis, seeds, taxonomy

## Abstract

Since the early 20th century, *Iris maackii* (Iridaceae) has been considered a synonym of *I. laevigata*, a synonym of *I. pseudacorus*, or an accepted species. The current concept of *I. maackii* in the literature and databases is often applied to yellow-flowered plants with prominently veined rosette leaves, which are diagnostic features of *I. pseudacorus* growing in Northeast Asia. Therefore, the objective was to clarify the taxonomic identity of *I. maackii*. This study is based on a critical examination of the literature, on the observed morphological characters in the holotype of *I. maackii*, and on a morphological comparison of *I. maackii* with living plants of *I. laevigata* and *I. pseudacorus*. Additionally, a morphometric comparison of the seed characters was carried out to clarify the morphological distinction among *I. maackii*, *I. laevigata*, and *I. pseudacorus*. A careful study demonstrated that the rosette leaf texture and the morphology of the flowering stem, fruit, and seeds of *I. maackii* are identical to or within the variation range of *I. laevigata*. Thus, *I. maackii* is morphologically non-distinct from *I. laevigata* and should be recognized as a taxonomic synonym of the latter. An image of the holotype of *I. maackii* is provided along with detailed illustrations of *I. laevigata* and *I. pseudacorus*.

## 1. Introduction

**Figure 1:**
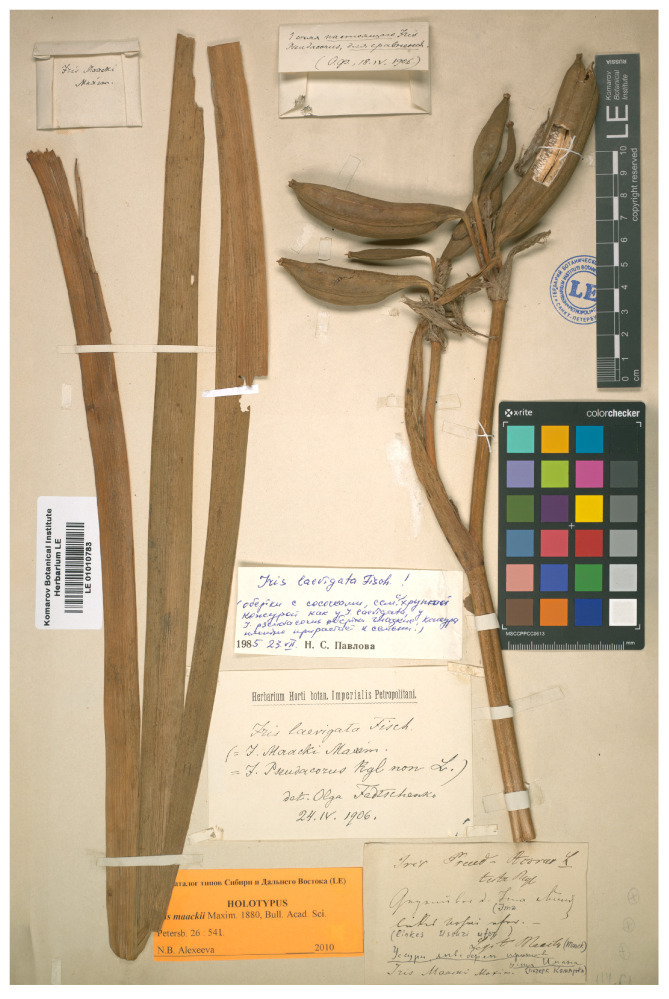
Holotype of *Iris maackii* (LE01010783) (included with the permission of the curator).

Richard Maack, an explorer and naturalist, took part in an expedition up the Ussuri River in June–August 1859 [1]. The botanical material from that expedition was treated by Eduard August von Regel in St. Petersburg [2]. In particular, Maack collected a fruiting specimen (Figure 1) from marshes on the Chinese left bank of the Ussuri River upstream of Shang-Ong (currently known as Hutou, northeastern Heilongjiang Province, China), opposite the mouth of the Iman River (currently known as the Bol’shaya Ussurka River, Primorsky Krai, Russia), on 15 July (27 July, according to the new calendar), 1859 [1]. Originally, Regel [2] (p. 148) identified this specimen as *Iris pseudacorus* L. However, Carl Johann Maximowicz described *I. maackii* Maxim. on the basis of Maack’s specimen [3].

The usage of the name *I. maackii* varies in the literature and databases. In fact, after being described, it was considered a synonym of *I. laevigata* Fisch. [4,5,6,7,8,9,10,11,12,13], or as a synonym of *I. pseudacorus* [14]. Currently, *I. maackii* is considered an accepted species native to Northeast Asia [15,16,17,18,19,20,21,22,23,24].

**Figure 2:**
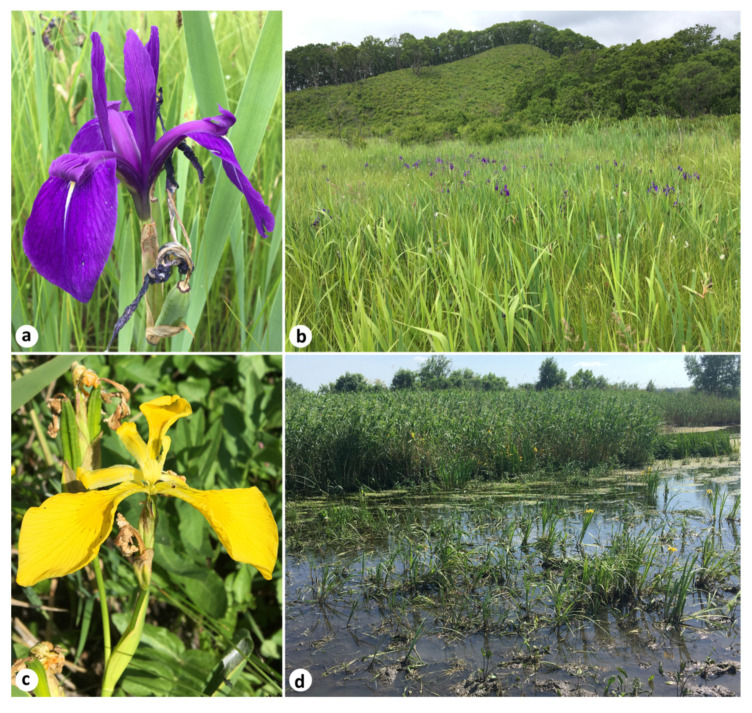
Images of the *Iris* species studied: a flower (**a**) and a habitat (**b**) of *I. laevigata* on a floating mat near Rudnev Bay, Primorsky Krai, Russia (42°55′10″ N 132°28′40″ E); a flower (**c**) and a habitat (**d**) of *I. pseudacorus* in the Tuzlov River, Rostov Oblast, Russia (47°28′15″ N 39°27′59″ E). Photos by the author.

*Iris laevigata* and *I. pseudacorus* are ornamental, wetland-associated, herbaceous perennials belonging to *I.* ser. *Laevigatae* (Diels) G.H.M.Lawr., according to the conservative taxonomy of *Iris* [8,9,25,26]. To the best of my knowledge, the blue-flowered *I. laevigata* (Figure 2a,b) is native to Northeast Asia, i.e., to the Russian Far East, northeastern China (Heilongjiang and Jilin provinces), the Korean Peninsula, and Japan (Hokkaido and Honshu islands). The natural distribution of *I. pseudacorus*, long known as the “yellow iris” and “yellow flag” (Figure 2c,d), covers Europe and extends to Western Siberia, Western Asia, and the northern fringe of Africa [27,28].

Regarding *I. pseudacorus*, it is necessary to pay special attention to some of its biological characteristics. On the one hand, due to the ability of *I. pseudacorus* to remove pollutants from water [27,29,30,31] and soil [32], it has been suggested to be used as an available and economically efficient species for phytoremediation. On the other hand, since *I. pseudacorus* has been extensively cultivated and naturalized, it is becoming highly invasive in North America, in the southern half of South America, southern South Africa, southeastern Australia, and New Zealand [21,33]. However, *I. maackii* is currently illustrated with images of *I. pseudacorus* and, therefore, these taxa are actually considered to be identical [34,35,36,37,38,39]. For this reason, the study of the morphological characters of *I. maackii*, as determined by its nomenclatural type, will contribute to the understanding of its taxonomy. This circumstance can undoubtedly improve the monitoring of the species’ invasion and help adjust the biocontrol program for *I. pseudacorus* [40,41,42].

This study aims to clarify the taxonomic identity of *I. maackii* in order to disentangle the confusion around this name. A comparison of rosette leaves, flowering stems, fruits, and seed morphology among *I. maackii*, *I. laevigata*, and *I. pseudacorus*, including data from the literature and field surveys, is presented. Detailed morphological illustrations of *I. laevigata* and *I. pseudacorus* based on complete material collected by the author are provided.

## 2. Materials and Methods

### 2.1. Plant Material and Morphological Study

*Iris maackii* was examined based on a single specimen (LE01010783!; Figure 1) that is a holotype of the name, consisting of two rosette leaf fragments and the upper part of the flowering stem, bearing mainly immature fruit. For plant morphology, this specimen was re-examined and 20 morphological characters, including 14 quantitative and 6 qualitative, were selected. These characters are listed in detail in Table 1. The author collected a total of 90 individuals of *I. laevigata* from a wild locality in the vicinity of Shtykovo Village (43°21′35″ N 132°22′1″ E, Primorsky Krai, Russia) on 27 June 2021 (Table S1). From 22 July to 3 August 2021, 63 individuals of *I. pseudacorus* were measured directly in the living collection of the Botanical Garden-Institute (BGI, Vladivostok, Russia). The measurements were taken during fruiting.

For the seed morphology, material from eight collection sites was used (Table 2). The study was conducted on mature seeds. The seeds of *I. maackii* were taken from one of the locules in the only mature fruit of LE01010783. For *I. laevigata* and *I. pseudacorus*, seeds were collected from different individuals for each site. The seeds of *I. laevigata* were from three wild localities in Russia, one of which is located near Lake Baikal, from where the species was described [43], and from two localities in Primorsky Krai. The seeds of *I. pseudacorus* were from the living collection of the BGI, Vladivostok, and also from two localities in Sakhalin Island (originally identified as *I. maackii*), one of which is indicated in references [16,44], and from a native population in the Don River delta.

The terminology used in the descriptions follows reference [45]. For the taxonomy, the *Shenzhen Code* (hereafter, ICN [46]) was consulted. Relevant literature, including the protologue of *I. maackii* [3], was also analyzed. The herbarium codes follow *Index Herbariorum* [47].

**Table 2:** Collection site data for seeds of the *Iris* species studied.

No.	Species	Origin	Voucher (*)
1	*I. maackii*	China, Ussuri River	*R. Maack* (LE01010783, holotype)
2	*I. laevigata*	Buryatia, Lake Baikal, near the Vydrinaya River estuary, 51°29′29.6″ N 104°50′39.3″ E	*Yu.N. Pochinchik* (VBGI)
3		Primorsky Krai, Bolshaya Ussurka River, Roshchino Village, 45°53′12.6″ N 134°50′48.3″ E	*L.M. Pshennikova* (VBGI)
4		Primorsky Krai, Khasansky District, Cape L’va, 42°41′60.0″ N 131°14′12.3″ E	*E.A. Chubar* (VBGI)
5	*I. pseudacorus*	Primorsky Krai, Vladivostok, BGI FEB RAS, 43°13′27″ N 131°59′38″ E	*E.V. Boltenkov* (VBGI, cult.)
6		Sakhalin Island, 3 km south of Shebunino Village	*s. coll.* (No. 2086, sub *I. maackii*) **
7		Sakhalin Island, Dolinsk	*s. coll.* (No. 2066, sub *I. maackii*) **
8		Rostov Oblast, Don River delta, 47°07′49.3″ N 39°28′07.7″ E	*A.N. Shmaraeva* (RWBG)

The collection site No. 6 was mentioned in references [16,44]; all sites of *I. laevigata* and *I. pseudacorus* are from Russia. * Herbarium codes follow *Index Herbariorum* [47]. ** Seed laboratory, Botanical Gardens of Peter the Great, Komarov Botanical Institute RAS (St. Petersburg, Russia).

### 2.2. Morphometric Analysis

The morphometric analysis was based on four seed characters (see Table 1), and used 50 seeds from each collection site (Table 2 and Table S2). All the statistical analyses were performed in the R software [48], version 4.1.2 [49]. The data were evaluated by one-way analysis of variance (ANOVA). After multiple statistical testing, the calculated *p*-values were adjusted using the procedure proposed by Benjamini and Hochberg [50]. To test the ANOVA assumptions, the Shapiro–Wilk test for the normality of the distribution [49] and Levene’s test for the homogeneity of the variance [51] were performed. The effect size (*η*2) for ANOVA and Cohen’s d for the difference in the means were calculated using the respective functions of the R add-on package “lsr”, version 0.6.1 [52]. If the ANOVA showed a statistically significant difference among species, then subsequent pairwise comparisons were made using Dunnett’s many-to-one test [53]. The data for *I. maackii* were used as a control. The inequality of variance was taken into account by using the heteroscedastic consistent covariance estimation provided in the R add-on package “sandwich”, version 2.3.0 [54,55]. The differences between the mean values of each collection site (Table 2) and the control were considered statistically significant at a *p*-value < 0.05.

Finally, a principal component analysis (PCA) [56] was performed on the morphometric parameters of the seeds, i.e., the L, W, T, and L/W ratio (see Table 1), to visualize the distribution of species over the space of the quantitative multivariate data and to assess their delimitation. For the PCA analysis, the built-in function *prcomp* was used, and the results of the analysis were extracted and visualized using the respective functions of the *factoextra* R package [57]. In the PCA scatter plot, only the first (PC1) and second (PC2) principal components were considered to represent the data.

## 3. Results

**Figure 3:**
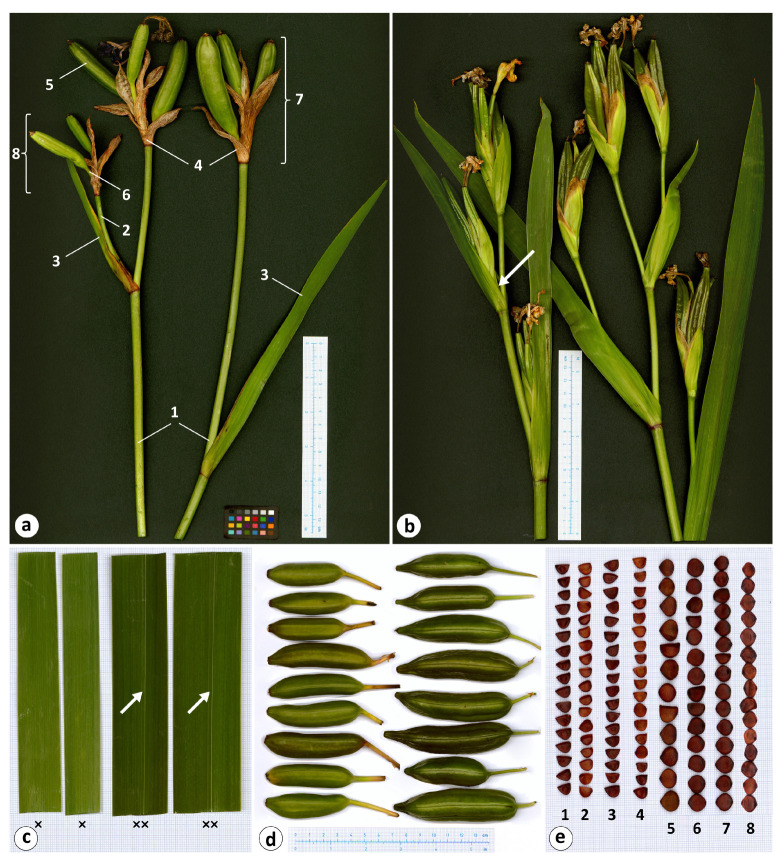
Morphological characters of *Iris laevigata* and *I. pseudacorus*: (**a**) flowering stems of *I. laevigata* (marks are as follows: 1, stem; 2, lateral shoot; 3, upper cauline leaf; 4, outer bract; 5, fruit; 6, pedicel; 7, terminal cluster; 8, lateral cluster); (**b**) flowering stems of *I. pseudacorus* (arrow indicates the inconspicuous lateral shoot); (**c**) a middle part of the rosette leaves (×, *I. laevigata*; ××, *I. pseudacorus*; arrows indicate the prominent midrib); (**d**) fruit (left row, *I. laevigata*; right row, *I. pseudacorus*); and (**e**) seeds (1–8 are collection site numbers; see Table 1). Photos by the author. Images were taken using an ObjectScan 1600 scanner (Microtek International Inc., Taiwan).

The selected characters of the species under study are shown in Figure 3 and Table 3 (also see Table S1). The holotype of *I. maackii* and the *I. laevigata* individuals were found to have no more than one lateral cluster per flowering stem, while in *I. pseudacorus*, there were usually more than one, and up to four, lateral clusters (Figure 1 and Figure 3a,b). *Iris maackii* and *I. laevigata* had smoothed rosette leaves that lacked the prominent midrib, a taxonomic feature of *I. laevigata*, with an average width of 1.7 cm; in *I. pseudacorus*, these leaves were prominently ribbed and broader, on average, by 60%, to 4.4 cm wide (Figure 1 and Figure 3c). The shoot of the upper lateral cluster was conspicuous and comparatively long in *I. maackii* and *I. laevigata*; in *I. pseudacorus*, it was usually inconspicuous or shorter than in *I. laevigata* (Figure 3a,b and Table 3). In *I. maackii* and *I. laevigata*, the upper cauline leaf was usually longer than in *I. pseudacorus* (Figure 3a,b). The bracts of *I. laevigata* were dry during fruiting; in *I. pseudacorus*, they were green. The number of fruit per stem was no greater than seven in *I. maackii* and *I. laevigata*, and they had smoothed surfaces and were obtuse at the apex; in *I. pseudacorus*, they were numerous (on average, 10), three-angled, and conspicuously beaked at the apex (Figure 3d). All three species shared the following characters: the bract and pedicel length, the number of fruit per terminal cluster and per upper lateral cluster, and the length and width of the fruit, which were oblong, cylindrical, and obtuse at the base (Figure 1 and Figure 3, Table 3).

The seed characters of the species under study are presented in Table 4 (also see Table S2). Morphologically, the seeds from all collection sites (Table 2) were brown, glossy, and flattened with a smooth surface and a more or less fragile testa (Figure 3e). The seed shape was oblong (L/W ratio: 1.4) or D-shaped in *I. maackii*; oblong (L/W ratio: 1.3–1.4), D-shaped, or, rarely, almost round in *I. laevigata*; in *I. pseudacorus*, the seed shape was almost round (L/W ratio: 0.9–1.1), predominantly subacute, or, much rarer, rounded at the chalaza. The seeds of *I. maackii* were similar in size to those of *I. laevigata* (Table 4). The seed length in *I. laevigata* ranged from 4.5 to 7.5 mm, the width from 3.8 to 6.6 mm, and the thickness from 1.4 to 3.6 mm. In *I. pseudacorus*, the values of these characters were greater: the seed length ranged from 5.6 to 9.6 mm, the width from 5.2 to 10.3 mm, and the thickness from 1.7 to 4.7 mm.

**Figure 4:**
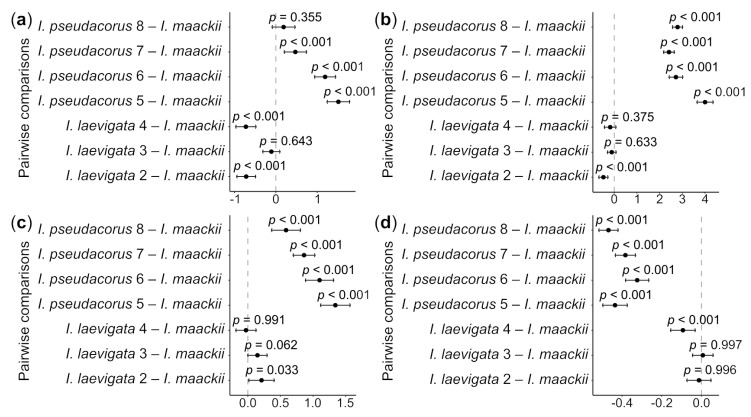
Whisker plots showing the results of Dunnett’s test of differences in the mean values of length (**a**), width (**b**), thickness (**c**), and L/W ratio (**d**) between the *Iris laevigata* and *I. pseudacorus* collection sites (see Table 2) and *I. maackii*, selected as a control. The results of the test are presented as *p*-values, differences between the mean values of each experimental group (black dots), and 95% confidence intervals of these differences (whiskers) for each pairwise comparison. X-axis is differences in means. Dash-dotted line indicates zero difference. See Table S3 for more details.

A statistically significant difference in all the morphometric parameters of the seeds was observed between the collection sites of *I. laevigata* and *I. pseudacorus* (see Table 1) and *I. maackii* (Figure 4; for ANOVA results, see Table S3). In particular, the seeds from sites 2 and 4 were approximately 10% shorter than those of *I. maackii*, with this difference being statistically significant (*p* < 0.001); the difference in the mean seed length between site 3 and *I. maackii* was 1.5%, being statistically non-significant (*p* > 0.05). The seeds from sites 5–7 were approximately 6.8–21.9% longer than the seeds of *I. maackii*, with this difference being statistically significant (*p* < 0.001). The difference in the seed length between site 8 and *I. maackii* was no greater than 2.7% and was statistically non-significant (*p* > 0.05).

No statistically significant differences in the seed width and thickness were found between collection sites 3 and 4 and *I. maackii* (*p* > 0.05), while there was a small, approximately 9.4%, but statistically significant (*p* < 0.001), difference between site 2 and *I. maackii*. There was no difference in the L/W ratio between sites 2 and 3 and *I. maackii* (*p* > 0.05). However, a small, approximately 6.8%, but statistically significant (*p* < 0.001), difference was found in the L/W ratio between site 4 and *I. maackii*. For *I. pseudacorus*, the mean seed width was approximately 37% greater, the seed thickness approximately 33.5% greater, and the L/W ratio approximately 41% smaller than for *I. maackii* and these differences were statistically significantly different (*p* < 0.001).

**Figure 5:**
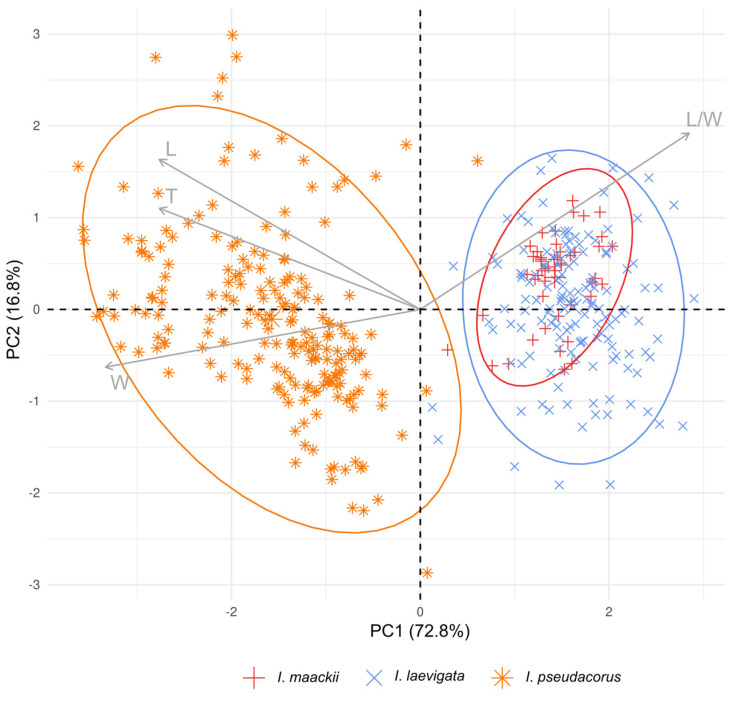
Principal component analysis of the morphometric parameters of seeds from *Iris maackii* (red), *I. laevigata* (blue), and *I. pseudacorus* (orange). Ellipses show 95% high-density regions for normal distributions representing two groups. Arrows indicate contribution of each morphometric parameter. The codes of the morphological characters of seeds are as follows: L, length; W, width; T, thickness; and L/W, length-to-width ratio. See Table S4 for more details.

In a PCA scatter plot (Figure 5), the first two principal components explained 89.6% of the total variance and revealed two distinct groups, corresponding to *I. laevigata* and *I. pseudacorus*. The first principal component (PC1) explained 72.8% of the total variance and contributed to discriminating the species (Figure 5; also see Table S4). Based on their correlation with the PC1 axis, the L, W, and T morphometric parameters were related to *I. pseudacorus* on the left side. In contrast, the L/W ratio was more significant for *I. maackii* and *I. laevigata*, which completely overlapped on the right side.

## 4. Discussion

### 4.1. What Is Iris maackii from Northeast Asia According to the Literature?

The taxonomic history of *I. maackii* began with a single fruiting specimen (Figure 1) collected by Maack from the middle reaches of the Ussuri River, in an area of present-day China, which was originally identified as *I. pseudacorus* by Regel [2]. Maximowicz came to the conclusion that Maack’s specimen was not *I. pseudacorus* in the terms of Linnaeus [58] (p. 38), because the leaves lacked the prominent midrib and, thus, the new species *I. maackii* Maxim. was described [3]. In addition, the mention of *I. pseudacorus* from Siberia by Regel [2] was indirectly related to references [59,60], according to which this species occurred in the vicinity of Selenginsk, Republic of Buryatia, and near Lake Baikal, Russia. It was rightly noted [4,61] that *I. pseudacorus* did not occur in Siberia or Manchuria, and its mention by Gmelin [59] (p. 31, No. 29) referred to *I. laevigata*, which had been described based on plants from the Baikal region and Dahuria [43].

Komarov noted that *I. maackii* had never been found in the type locality after Maack [62]. Fedtschenko reported that the bract texture and fruit shape of *I. maackii* were the same as those of *I. laevigata*, and regarded *I. maackii* as a synonym of *I. laevigata* [4]. Ivan Shishkin, a florist of the Far Eastern Branch of the USSR Academy of Sciences, made six expeditions in 1927–1929 with the aim to extend the floristic knowledge of the Iman River and surroundings [7]. In particular, he came to the conclusion that only *I. laevigata*, including *I. pseudacorus* in the terms of Regel [2], and *I. maackii* in the terms of Komarov [62] could be found in this area. Thus, the name *I. maackii* was synonymized with *I. laevigata* [5,8,9,10].

On the other hand, *I. maackii* was indicated for Sakhalin Island and the Kuril Islands and characterized as a plant with a prominently veined mid-rib and yellow flowers [44,63,64,65], although these are the diagnostic features of *I. pseudacorus*. However, most recent authors state that *I. maackii* from the Russian Far East is *I. pseudacorus*, which was introduced there and naturalized [11,66,67,68]. Apparently, Russian settlers in this region introduced *I. pseudacorus* as a bactericidal rather than an ornamental plant. The essential oils from the rhizomes of *I. pseudacorus* were shown to have antimicrobial activity [69,70]. Moreover, this plant can reduce the number of coliform bacteria by 50% and *Salmonella* by 70% in wastewater [27]. In addition, the Far Eastern and European plants of *I. pseudacorus* have the same chromosome number, 2*n* = 34 [66] (p. 127), and are not different in the noncoding regions of plastid DNA [26].

In addition, *I. maackii* was indicated to grow in the Liaoning, Heilongjiang, and Jilin provinces of northeastern China [18,25,71,72,73,74,75]. Zhao noted that the characteristics of *I. maackii* from Liaoning Province are similar to *I. pseudacorus*, and, therefore, he doubted that *I. maackii* was a true species [76]. The authors of the *Flora of China* noted that further study was needed to determine whether or not *I. maackii* is separable from *I. pseudacorus* [25]. Rodionenko suggested that *I. maackii* from China was the adventive *I. pseudacorus* [28]. Recently, it has been confirmed that the plants from Liaoning Province cited as *I. maackii* do, in fact, belong to *I. pseudacorus*, while *I. laevigata* was not found in the province [77].

Thus, authors in the beginning of the 21th century treat *I. maackii* from Northeast Asia as *I. pseudacorus*. Notwithstanding the above, *I. maackii* is currently considered an accepted species native to northeastern China and the Russian Far East [15,16,17,18,19,20,21,22,23,24,34,36,37,38,39].

### 4.2. What Is Iris maackii vs. I. laevigata and I. pseudacorus According to Morphology?

An examination of the holotype of *I. maackii* (Figure 1) showed it to be identical to *I. laevigat*a in the rosette leaf width and texture, flowering stem branching, length of the upper lateral shoot, bract texture during fruiting, number of fruit per flowering stem, fruit shape, and size and shape of the seeds (Table 3 and Table 4, Figure 1 and Figure 3). In addition, after the characterization of 153 individuals of *I. laevigata* and *I. pseudacorus* for 15 morphological characters, it was found that these species could be differentiated from each other, especially in the rosette leaf texture, the flowering stem branching, and the fruit shape (Figure 3 and Table 3).

Among the species studied, the seeds varied in size and shape. By using Dunnett’s test, differences in the morphometric parameters of seeds were found between *I. maackii* and some of the collection sites of *I. laevigata* (Figure 4). However, these differences were no greater than 10% and were related to the origin of the seeds: the seeds of *I. maackii* were from a single available locule of the same fruit and, therefore, had similar sizes (Figure 3e), while the seeds of *I. laevigata* were from different individuals. In the PCA scatter plot, the characters of *I. maackii* completely overlapped with those of *I. laevigata* (Figure 5); the characters were taxonomically useful when the overlap was equal to or lower than a threshold of 25% [78].

Previously, it was reported that the seed characters of *I. laevigata* were almost identical to those of *I. pseudacorus* [79,80,81]. However, that finding is not in concurrence with the present study. An analysis of the morphometric parameters of seeds based on Dunnett’s test showed that *I. laevigata* and *I. pseudacorus* are distinct (Figure 4). In view of the results of the PCA analysis, the three taxa could unambiguously be separated into two distinct groups with clearly different features in their seed characters (Figure 5). In particular, it was confirmed that the plants listed in references [16,44] as *I. maackii* from the neighborhood of Shebunino Village, Sakhalin Island, belonged to *I. pseudacorus* (Figure 4 and Figure 5). Thus, the morphological differences between *I. laevigata* and *I. pseudacorus* also include seed characters such as the size and shape.

### 4.3. Taxonomic Treatment

Based on detailed morphological and morphometric comparisons among *I. maackii*, *I. laevigata*, and *I. pseudacorus*, two species are recognized in the present study, *I. laevigata* and *I. pseudacorus*. With regards to *I. maackii*, the author postulates that this name is a taxonomic synonym of *I. laevigata*. Information on the accepted species (highlighted in bold italics) is provided below.

***Iris laevigata*** Fisch., Index Seminum (St.Petersburg (Petropolitanus)) 5: 36, 1839.—Lectotype (designated by Alexeeva [82] (p. 417)): In paludibus ad Baicalem, [fl.], 1829, *Turcz*[*aninow*] *s.n*. Herb. C.F. Ledebour (LE01010777!).—http://rr.herbariumle.ru/01010777 (accessed on 13 August 2023).

=*Iris maackii* Maxim., Bull. Acad. Imp. Sci. Saint-Pétersbourg 26(3): 541, 1880.—Holotype: (China, Heilongjiang Province) (note handwritten by E. Regel): *Iris Pseud-Acorus* L. teste Rgl. Legit Maack; (note handwritten by C.J. Maximowicz): *Iris maackii* Maxim. Gegenüber d. [der] Ima Mündung linkes Ussuri uter, [fr.], (15 (27) July 1859); (note handwritten by V.L. Komarov): Уссури, левый берег прoтив устья Имана (Ussuri River, left bank opposite the mouth of the Iman River) (LE01010783!).—Figure 1.

=*Iris pseudacorus* auct. non L. [2,60].

***Iris pseudacorus*** L., Sp. Pl. 1: 38, 1753.—“*I. pseudacorus* var. *mandshurica* L.H.Bailey”, Man. Cult. Pl., ed. 2: 273, 1949, *nom. inval*. (Art. 38.1 of the ICN).—Lectotype (designated by Crespo [83] (p. 56)): (Specimen from a cultivated plant). *PseudoAcorus* 7, [fl.], s.d., *s. coll. s.n.* Herb. Linnaeus (LINN No. 61.7!).—https://www.linnean-online.org/805/ (accessed on 13 August 2023).

=*Iris maackii* auct. non Maxim. [15,16,25,44,63,64,65,71,72,73,74,75].

## 5. Conclusions

Since the early 20th century, the taxonomic identity of *Iris maackii* (Iridaceae) has been unclear, and there have been various speculations as to whether it is an independent species or not. Currently, in most databases, it is regarded as a distinct species native to northeastern China and the Russian Far East [17,18,19,20,21,22,23,24,34,36,37,38,39]. The present report provides a re-evaluation of the taxonomic identity of *I. maackii* based on a morphological study. In addition, an overview of the taxonomic history of *I. maackii*, based on numerous publications of scientists from 1861 to the present time, was conducted to establish its true identity. Since *I. maackii* is known on the basis of a single specimen (Figure 1), there was difficulty with the availability of material for the morphological comparison of this species with *I. laevigata* and *I. pseudacorus*, to which it is associated. However, as a result of a careful examination of the holotype of *I. maackii*, a total of 20 morphological characters were selected.

As argued in the present contribution, *I. maackii* is a taxonomic synonym of *I. laevigata* on the basis of a set of characters, including smoothed rosette leaves, one-branched flowering stems, an elongated shoot of the upper lateral cluster, dry bracts during fruiting, fruit that is smoothed and obtuse at the apex, and mostly oblong, D-shaped seeds. In addition, it is of equal importance that the species-specificity of the seed size and shape can be useful in the taxonomic differentiation of *I. laevigata* and *I. pseudacorus*. In order to avoid further confusion, it is here stated that the names *I. maackii* and *I. pseudacorus* must never be conflated. The present results confirm that the plants from northeastern China and the Russian Far East (viz. Sakhalin Island, Kuril Islands, and Primorsky Krai) indicated in the literature (e.g., [15,16,44]) and databases [17,18,19,20,21,22,23,24,34,36,37,38,39] as *I. maackii* should be considered *I. pseudacorus*. An important point is that *I. pseudacorus* is non-native in Northeast Asia and has become highly invasive in natural and artificial waterbodies in the Neotropics, Afrotropics, Neartic, and Australasia.

## Figures and Tables

**Table 1:** Morphological characters examined.

No.	Characters	Remarks
1	Rosette leaf width	Measured at the largest part of the widest rosette leaf
2	Rosette leaf texture	When dry, the surface of rosette leaves is finely ribbed, lacks a prominent midrib (smoothed), or has 1–2 large median veins generally running very close together, resembling in appearance a midrib (ribbed)
3	Stem branching	Flowering stem classified as simple, bearing only the terminal cluster (designated as 0), or branched, with lateral clusters (designated as 1, etc.)
4	Shoot length	Measured for the shoot of the upper lateral cluster
5	Cauline leaf length	Measured from the base to the apex of the upper cauline leaf
6	Bract length	Measured from the base to the apex of the outer bract of the terminal cluster
7	Bract texture	When fruiting, bracts dry or green
8	Pedicel length	Measured from the base of the terminal cluster to the ovary base of the first blooming flower
9	Fruit total	Number of fruit per stem
10	Fruit terminal	Number of fruit per terminal cluster
11	Fruit lateral	Number of fruit per upper lateral cluster
12	Fruit shape	Surface smoothed or angled, and characterized as sharply narrowed at the apex (obtuse) or conspicuously shortly beaked
13	Fruit length	Measured for the first fruit of the terminal cluster (using an Absolute Digimatic digital caliper, Mitutoyo, USA, to an accuracy of 0.1 mm)
14	Fruit width	
15	Seed shape	Inequilateral, tapering to hilum and chalaza, i.e., D-shaped or almost rounded
16	Seed color	Color was described in subjective terms
17	Seed length (L)	For seed characters, 50 samples from each collection site (see Table S2) were used (all were measured with the same сaliper)
18	Seed width (W)	
19	Seed thickness (T)	
20	Seed L/W ratio	The length-to-width ratio (L/W) provides additional data on the seed shape, i.e., degree of elongation along the hilum–chalaza axis (all calculations were manual)

**Table 3:** Comparative morphology of the *Iris* species studied.

No.	Characters	*I. maackii*	*I. laevigata*	*I. pseudacorus*
1	Rosette leaf width	1.8	1.7 (0.85–2.9)	3.0 (1.3–4.4)
2	Rosette leaf texture	Smoothed	Smoothed	Ribbed
3	Stem branching	1	0–1	1–4
4	Shoot length	10.5	9 (4.2–13)	4.7 (0.2–12.0)
5	Cauline leaf length	>16	18.7 (8.5–28.5)	10.8 (4.7–26.2)
6	Bract length	≥4.3	5.8 (3.8–10)	5.7 (4–9.8)
7	Bract texture	Consisting of remnants	Dry	Green
8	Pedicel length	2.0	2.1 (0.5–4.2)	2.6 (1.4–4.8)
9	Fruit total	7	4 (1–7)	10 (5–17)
10	Fruit terminal	4	4 (1–6)	4 (2–5)
11	Fruit lateral	3	1 (0–3)	2 (1–3)
12	Fruit shape	Smoothed; obtuse	Smoothed; obtuse	3-angled; beaked
13	Fruit length	8.7	6.3 (4–8)	7.6 (6–9.5)
14	Fruit width	1.8	1.5 (1.2–1.8)	1.9 (1.5–2.4)
15	Seed shape	Oblong, D-shaped	Mostly oblong, D-shaped, or almost round	Mostly almost round or D-shaped
16	Seed color	Brown	Brown	Brown

The descriptions of the characters are provided in Table 1. Data for *I. laevigata* and *I. pseudacorus* are presented as the mean (minimum–maximum); see supplementary raw data in Table S1 for more details. All measurements are in centimeters.

**Table 4:** Morphological characters of seeds from the *Iris* species studied.

No.	Species	Seed			
		Length (L)	Width (W)	Thickness	L/W Ratio
1	*I. maackii*	6.9 ± 0.5 (5.3–7.5)	5.0 ± 0.4 (3.7–6.5)	1.9 ± 0.3 (1.5–3.5)	1.4 ± 0.1 (1.1–1.6)
2	*I. laevigata*	6.2 ± 0.4 (4.5–6.9)	4.6 ± 0.4 (3.8–5.4)	2.1 ± 0.4 (1.5–3.6)	1.4 ± 0.1 (1.1–1.7)
3		6.8 ± 0.3 (6.1–7.5)	4.9 ± 0.3 (4.3–5.8)	2.1 ± 0.2 (1.6–2.5)	1.4 ± 0.1 (1.1–1.6)
4		6.2 ± 0.5 (5.2–6.9)	4.9 ± 0.6 (4.0–6.6)	1.9 ± 0.3 (1.4–2.8)	1.3 ± 0.1 (1.0–1.6)
5	*I. pseudacorus*	8.4 ± 0.6 (7.0–9.6)	9.0 ± 0.9 (6.3–10.3)	3.3 ± 0.5 (2.3–4.7)	0.9 ± 0.1 (0.7–1.4)
6		8.1 ± 0.5 (6.9–9.2)	7.8 ± 0.7 (5.6–8.8)	3.0 ± 0.5 (2.2–4.5)	1.1 ± 0.1 (0.9–1.4)
7		7.4 ± 0.6 (6.4–8.9)	7.5 ± 0.5 (5.2–8.4)	2.8 ± 0.3 (2.2–3.5)	1.0 ± 0.1 (0.9–1.5)
8		7.1 ± 0.6 (5.6–8.7)	7.8 ± 0.5 (6.8–8.5)	2.5 ± 0.5 (1.7–3.9)	0.9 ± 0.1 (0.7–1.1)

The descriptions of the characters are provided in Table 1. The collection site numbers (No.) correspond to those in Table 2. Data are presented as the mean ± standard deviation (minimum–maximum). See supplementary raw data in Table S2 for more details. All measurements are in millimeters.

## Data Availability

All data supporting reported results are presented as Supplementary Materials.

## Supplementary Materials

The following supporting information can be downloaded at: https://www.mdpi.com/article/10.3390/plants12193349/s1, Table S1: Raw data of the morphological analysis of the *Iris* species studied (the numbers of the morphological characters correspond to those in Table 1); Table S2: Raw data of the morphological analysis of seeds from the *Iris* species (the codes of the morphological characters are provided in Table 1; for the collection site numbers, i.e., 1–8, see Table 2); Table S3: Results of ANOVA and pairwise comparisons between the mean values for each collection site (see Table 1) and the control (*I. maackii*) using Dunnett’s many-to-one test for the morphometric parameters of seeds; Table S4: Results of the principal component analysis of the morphometric parameters of seeds.
