# Supplementary material for: Resolving the Puzzle of Iris maackii (Iridaceae): A Morphological Insight into Its Taxonomy
Source: Plants (Basel). 2023 Sep 22;12(19):3349. doi: 10.3390/plants12193349 (PMC10574458; doi:10.3390/plants12193349)
Supplement: Supplementary file 1 [file plants-12-03349-s001.zip › Table S3.pdf]

Table S3. Results of ANOVA and pairwise comparisons between the mean values for each collection site (see Table 1) and the control (*I. maackii*) by the Dunnett's many-to-one test for the morphometric parameters of seeds.

Pairwise comparisons	MD	*S*e	*t*-value	*p*-value	LM	UM
**Seed length** (L); ANOVA: F (7,392) = 130.77, $p < 0.0001$, $\eta^2 = 0.70$						
I. laevigata-2 – *I. maackii* = 0*	–0.71600	0.08864	–8.078	< 0.001	–0.94948	–0.48252
I. laevigata-3 – *I. maackii* = 0	–0.10800	0.08096	–1.334	0.643	–0.32127	0.10527
I. laevigata-4 – *I. maackii* = 0	–0.72400	0.09477	–7.640	< 0.001	–0.97362	–0.47438
I. pseudacorus-5 – *I. maackii* = 0	1.51200	0.10585	14.285	< 0.001	1.23319	1.79081
I. pseudacorus-6 – *I. maackii* = 0	1.19200	0.09880	12.064	< 0.001	0.93174	1.45226
I. pseudacorus-7 – *I. maackii* = 0	0.47200	0.10462	4.512	< 0.001	0.19642	0.74758
I. pseudacorus-8 – *I. maackii* = 0	0.18800	0.10772	1.745	0.355	–0.09576	0.47176
**Seed width** (W); ANOVA: F (7,392) = 496.39, $p < 0.0001$, $\eta^2 = 0.90$						
I. laevigata-2 – *I. maackii* = 0	–0.47400	0.07991	–5.932	< 0.001	–0.68607	–0.26193
I. laevigata-3 – *I. maackii* = 0	–0.10600	0.07623	–1.391	0.633	–0.30831	0.09631
I. laevigata-4 – *I. maackii* = 0	–0.17600	0.10043	–1.753	0.375	–0.44252	0.09052
I. pseudacorus-5 – *I. maackii* = 0	4.00400	0.13806	29.002	< 0.001	3.63761	4.37039
I. pseudacorus-6 – *I. maackii* = 0	2.72000	0.11812	23.027	< 0.001	2.40652	3.03348
I. pseudacorus-7 – *I. maackii* = 0	2.41400	0.09362	25.785	< 0.001	2.16554	2.66246
I. pseudacorus-8 – *I. maackii* = 0	2.79200	0.08827	31.629	< 0.001	2.55773	3.02627
**Seed thickness**; ANOVA: F (7,392) = 87.09, $p < 0.0001$, $\eta^2 = 0.61$						
I. laevigata-2 – *I. maackii* = 0	0.21000	0.07508	2.797	0.0331	0.01110	0.40890
I. laevigata-3 – *I. maackii* = 0	0.14800	0.05762	2.569	0.0618	–0.00464	0.30064
I. laevigata-4 – *I. maackii* = 0	–0.02800	0.06024	–0.465	0.9981	–0.18760	0.13160
I. pseudacorus-5 – *I. maackii* = 0	1.34200	0.08688	15.447	< 0.001	1.11185	1.57215
I. pseudacorus-6 – *I. maackii* = 0	1.10000	0.08318	13.225	< 0.001	0.87964	1.32036
I. pseudacorus-7 – *I. maackii* = 0	0.86200	0.06432	13.402	< 0.001	0.69160	1.03240
I. pseudacorus-8 – *I. maackii* = 0	0.58600	0.08499	6.895	< 0.001	0.36085	0.81115
**L/W ratio**; ANOVA: F (7,392) = 169.05, $p < 0.0001$, $\eta^2 = 0.75$						
I. laevigata-2 – *I. maackii* = 0	–0.012318	0.023652	–0.521	0.996	–0.074701	0.050065
I. laevigata-3 – *I. maackii* = 0	0.007178	0.020165	0.356	1.000	–0.046007	0.060364
I. laevigata-4 – *I. maackii* = 0	–0.093207	0.024026	–3.879	< 0.001	–0.156578	–0.029837
I. pseudacorus-5 – *I. maackii* = 0	–0.435392	0.023964	–18.169	< 0.001	–0.498598	–0.372186
I. pseudacorus-6 – *I. maackii* = 0	–0.323667	0.022796	–14.199	< 0.001	–0.383792	–0.263542
I. pseudacorus-7 – *I. maackii* = 0	–0.383088	0.019862	–19.288	< 0.001	–0.435475	–0.330701
I. pseudacorus-8 – *I. maackii* = 0	–0.468369	0.018903	–24.777	< 0.001	–0.518227	–0.418511

MD, difference in means; *S*e, standard error of mean; LM, lower margin of 95% confidence interval; UM, upper margin of 95% confidence interval. * Testing the hypothesis that the difference between the mean values of the variable in a pairwise comparison is zero.
