# Supplementary material for: Resolving the Puzzle of Iris maackii (Iridaceae): A Morphological Insight into Its Taxonomy
Source: Plants (Basel). 2023 Sep 22;12(19):3349. doi: 10.3390/plants12193349 (PMC10574458; doi:10.3390/plants12193349)
Supplement: Supplementary file 1 [file plants-12-03349-s001.zip › Table S4.pdf]

**Table S4.** Results of the principal component analysis of the morphometric parameters of seeds.

| **Parameter** | **Components** | | |
|---|---|---|---|
| | **PC1** | **PC2** | **PC3** |
| Length (L) | −0.471 | **0.579** | **0.563** |
| Width (W) | **−0.568** | −0.223 | 0.233 |
| Thickness (T) | −0.471 | 0.390 | **−0.791** |
| L/W ratio | 0.485 | **0.681** | 0.051 |

Scores of the variables for the first three main axes are shown. The variables that contributed most for the axes are highlighted in bold.
